# The important role of palpation in the examination of solitary skin lesions: mnemonic of three T’s

**DOI:** 10.1093/skinhd/vzaf115

**Published:** 2026-01-28

**Authors:** Alexander Salava

**Affiliations:** Department of Dermatology, Venereology and Allergology, University Hospital Helsinki and University of Helsinki, Helsinki, Finland

## Abstract

The author describes an easy applicable three-step teaching method to increase learning outcomes in dermatology education and physical examination: the mnemonic of three T’s. It summarizes the three most important aspects of palpation in a methodologically simple to learn and stepwise manner and can be used to systematize teachings, indicating the important role of palpation, and to enhance communication and student motivation.

Palpation plays an important role in the examination of patients, especially of solitary skin lesions. Teaching students proper skills of physical examination should be regarded as an essential part of any dermatology curriculum and palpation can be viewed as an extension of detecting and recognizing cutaneous findings.^1^ However, in contrast to visual assessment (inspection), which in dermatology traditionally follows systematic approaches (primary lesion, pattern recognition, distribution and body site), palpation cannot be learned based on clinical pictures, digital self-learning tools or presentations;^2^ it has to be trained practically as a procedural skill and is therefore mainly taught bedside, on individual patients, during simulation or in similar settings. This causes major educational challenges regarding ample repetitions and sufficient exploration of a variety of lesions. It is also time- and resource-consuming and there have been recent signs of decreasing clinical practice training of medical students.^3^

For many years, the author has used an easily applicable three-step method to increase learning outcomes during the teaching of physical examinations of solitary skin lesions, the mnemonic of three T’s. It summarizes the three most important aspects of palpation in a methodologically simple-to-learn and stepwise manner (Table 1). The first T stands for texture (surface structures), which should be evaluated with the palmar sides of the fingertips. A typical clinical example would be the rough, stuck-on feeling in actinic keratosis, which is often better felt than assessed visually (Figure 1a). The second T corresponds to tissue (consistency), which can be estimated by placing the cutaneous lesion between the thumb and index fingers and pressing or squeezing it (tweezer grip). A good example is the characteristic firm rubber-like composition of keloid scars (Figure 1b). It is also frequently used to clarify boundaries of skin cancer (e.g. basal cell carcinoma) if the skin is stretched outward. The third T represents traction and manoeuvrability (mobility). In this last step, the lesion is moved horizontally to its base and underlying tissue and so possible attachments, or depth expansion, can be distinguished. It may also be helpful to move adjacent muscles, tendons or joints to recognize fixations. A practical example would be a movable soft lipoma and the possibility of raising a skin fold above it (Figure 1c).

**Figure 1:**
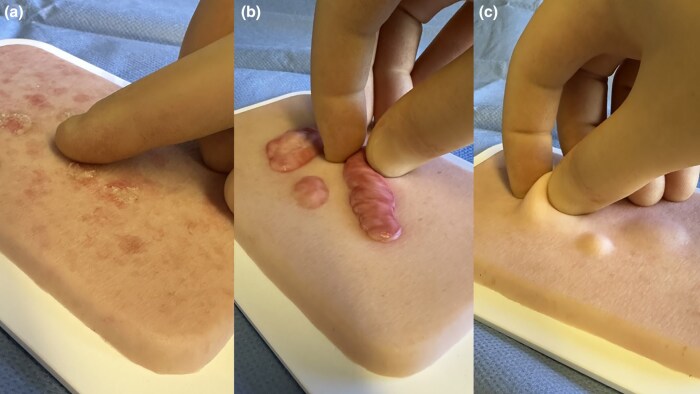
Teaching the important role of palpation in the examination of solitary skin lesions: the mnemonic of three T’s: (a) texture (surface structures), (b) tissue (consistency and possible symptoms) and (c) traction (movability or fixation to underlying structures). The fingertips are best used to assess texture, and the thumb and index fingers (tweezer grip) are used to evaluate consistency and movability. The mnemonic of three T’s may be used in bedside teaching or simulation-based training. The figure depicts the practice of palpation based on the mnemonic’s step wise manner using 3D silicone models (Medical FX^®^, Kirchlinteln, Germany): (a) actinic keratosis; (b) keloid scar; (c) lipoma.

**Table 1:** Use and practice of mnemonic of the three T’s, with clinical examples; the mnemonic can help to systematize and enhance teaching physical examinations of solitary skin lesions, and summarizes the three most important aspects of palpation in a methodologically simple-to-learn and stepwise manner

Practice	Assessment	Clinical examples
**T**exture (feel the lesion with fingertips)	Surface structures (e.g. rough, smooth, succulent, velvety, verrucous) Scaling and hyperkeratosis (e.g. tightly attached, sharp) Ulceration, demarcation (e.g. irregular or regular edges) Temperature (e.g. warm, temperate)	Actinic keratosis: rough and sharp^4^ (Figure 1a) Molluscum contagiosum, intradermal naevus: smooth Lipoma, epidermal inclusion cyst, lymph node: smooth Pyogenic granuloma, angiosarcoma, cutaneous metastases: succulent, soft Wart or condyloma: verrucous or velvety
**T**issue (press, squeeze or stretch the lesion between thumb and index fingers, ‘tweezer grip’, apply a light source directly to the lesion, e.g. pen torch)	Consistency (e.g. soft, firm, hard, fluctuation) Borders of the lesion (e.g. by stretching it) Pulsation Tenderness (e.g. pain, paraesthesia) Transillumination (e.g. translucent)	Keloids, hypertrophic scars: firm, rubber-like^5^ (Figure 1b) Lymph node: firm, tender Lipoma, epidermal inclusion cyst: soft (Figure 1c) Metastases: hard, like stones Boil: fluctuation, tenderness Vascular aetiology: pulsation Nodular basal cell carcinoma: clear borders when stretched Dermatofibroma: squeezing causes indentation, ‘dimple sign’^6^ Mucocoele and other fluid-filled lesions will allow light to pass through
**T**raction (hold the lesion between thumb and index fingers and move it horizontally to its base and underlying tissue, apply gentle pressure)	Attachment to underlying structures (e.g. freely movable or fixed in regard to its base) Mobility (e.g. movability in regard to its place) Irreducibility (e.g. can or cannot be reduced, can be pressed into the skin, reappearance when released) Fixation to tendons, muscles, joints, etc.	Lipoma: freely movable to its place and base Epidermal inclusion cyst: fixed to its place but freely movable to its base Ganglion cyst: fixed to its base and place Infiltrating squamous cell carcinoma: fixed to underlying structures, e.g. fascia^7^ Metastatic lymph nodes: fixed together Neurofibroma: retractable into the skin when pressed and reappears upon release, ‘buttonhole sign’ Inguinal hernia: can be reduced Tendon calcinosis: moves with tendons Bone tumours, e.g. exostosis: firmly fixed to its base

The mnemonic can be used to systematize the teaching of palpation during physical examination of solitary skin lesions. It is particularly useful when there is limited time, for example in bedside or simulation-based education. The teacher can underline the important role palpation plays in the process of physical examination and also clarify what additional information it may provide. In addition, it can be used to show that the tactile perception of touch may contribute to a better doctor–patient relationship and patients feeling that they have been properly examined.^8^ The mnemonic can also be used as an easily available memory aid to improve communication between students and the teacher. In the author’s experience, it may be useful in a wide range of settings (e.g. bedside, simulation and three-dimensional models) and is likely to increase student motivation and enhance teaching of clinical examination – the most important skill in diagnosing skin conditions.^9^

## Data availability

There were no data collected or created.
